# A Change of Position

**Published:** 1887-02

**Authors:** 


### A Change of Position.

One of the most pleasant and satisfactory features of the recent meeting of the first District Dental Society, of New York, was the banquet given on Wednesday evening, the 19th. About one hundred and fifty invited guests were seated at the tables, which were prepared in superb style. All enjoyed the occasion to the full. The table was all, yes, more, than the most epicurian taste could desire. A number of toasts were given and responded to in a most happy manner; the flow of humor, wit, and wisdom was constant, and was highly enjoyed by all present.

The most remarkable speech of the evening was by Dr. N. W. Kingsley, in which he assumed a new position in regard to the Ninth International Medical Congress, and especially toward the Section on Dental and Oral Surgery. It is well known that Dr. Kingsley has for some time advocated, with his characteristic energy and enthusiasm, the holding in the near future an International Dental Congress; this, if carried out, according to the plan of some, at least, would very seriously affect and impair the success of the Section of Dental and Oral Surgery. This, in the estimation of a large proportion of the profession, was deemed very unfortunate. Dr. Kingsley, perceiving the trend of opinion and not wishing to occupy a position in antagonism to that so generally entertained, announced that he now abandoned the idea and advocacy of holding an *International Dental Congress;* at least, for the present, or at any time that would interfere with the success and best interest of the *Dental Section.* And, moreover, that he would give his hearty support and co-operation to make this *Section* the greatest possible success. He further expressed the hope and belief that those who heretofore have entertained views similar to his own would now give their support and co-operation to the Dental Section.

The remarks made, and the position taken by Dr. Kingsley, were received with marked demonstrations of satisfaction and approval, and add much to the indications of success for the Section; and to this end perfect harmony and co-operation are hoped for.

